# Non-human Primate Models to Investigate Mechanisms of Infection-Associated Fetal and Pediatric Injury, Teratogenesis and Stillbirth

**DOI:** 10.3389/fgene.2021.680342

**Published:** 2021-07-05

**Authors:** Miranda Li, Alyssa Brokaw, Anna M. Furuta, Brahm Coler, Veronica Obregon-Perko, Ann Chahroudi, Hsuan-Yuan Wang, Sallie R. Permar, Charlotte E. Hotchkiss, Thaddeus G. Golos, Lakshmi Rajagopal, Kristina M. Adams Waldorf

**Affiliations:** ^1^Department of Obstetrics & Gynecology, University of Washington, Seattle, WA, United States; ^2^Department of Biological Sciences, Columbia University, New York, NY, United States; ^3^Department of Global Health, University of Washington, Seattle, WA, United States; ^4^Elson S. Floyd College of Medicine, Washington State University, Spokane, WA, United States; ^5^Department of Pediatrics, Emory University School of Medicine, Atlanta, GA, United States; ^6^Yerkes National Primate Research Center, Emory University, Atlanta, GA, United States; ^7^Center for Childhood Infections and Vaccines of Children’s Healthcare of Atlanta and Emory University, Atlanta, GA, United States; ^8^Department of Pediatrics, Weill Cornell Medicine, New York, NY, United States; ^9^Washington National Primate Research Center, University of Washington, Seattle, WA, United States; ^10^Department of Comparative Biosciences, University of Wisconsin-Madison, Madison, WI, United States; ^11^Department of Obstetrics and Gynecology, University of Wisconsin-Madison, Madison, WI, United States; ^12^Wisconsin National Primate Research Center, University of Wisconsin-Madison, Madison, WI, United States; ^13^Department of Pediatrics, University of Washington, Seattle, WA, United States; ^14^Center for Global Infectious Disease Research, Seattle Children’s Research Institute, Seattle, WA, United States; ^15^Department of Obstetrics and Gynecology, Sahlgrenska Academy, University of Gothenburg, Gothenburg, Sweden

**Keywords:** nonhuman primate, teratogenesis, Zika virus, HIV, preterm birth, group B streptococcus, cytomegalovirus, listeria

## Abstract

A wide array of pathogens has the potential to injure the fetus and induce teratogenesis, the process by which mutations in fetal somatic cells lead to congenital malformations. Rubella virus was the first infectious disease to be linked to congenital malformations due to an infection in pregnancy, which can include congenital cataracts, microcephaly, hearing impairment and congenital heart disease. Currently, human cytomegalovirus (HCMV) is the leading infectious cause of congenital malformations globally, affecting 1 in every 200 infants. However, our knowledge of teratogenic viruses and pathogens is far from complete. New emerging infectious diseases may induce teratogenesis, similar to Zika virus (ZIKV) that caused a global pandemic in 2016–2017; thousands of neonates were born with congenital microcephaly due to ZIKV exposure *in utero*, which also included a spectrum of injuries to the brain, eyes and spinal cord. In addition to congenital anomalies, permanent injury to fetal and neonatal organs, preterm birth, stillbirth and spontaneous abortion are known consequences of a broader group of infectious diseases including group B streptococcus (GBS), *Listeria monocytogenes*, Influenza A virus (IAV), and Human Immunodeficiency Virus (HIV). Animal models are crucial for determining the mechanism of how these various infectious diseases induce teratogenesis or organ injury, as well as testing novel therapeutics for fetal or neonatal protection. Other mammalian models differ in many respects from human pregnancy including placentation, labor physiology, reproductive tract anatomy, timeline of fetal development and reproductive toxicology. In contrast, non-human primates (NHP) most closely resemble human pregnancy and exhibit key similarities that make them ideal for research to discover the mechanisms of injury and for testing vaccines and therapeutics to prevent teratogenesis, fetal and neonatal injury and adverse pregnancy outcomes (e.g., stillbirth or spontaneous abortion). In this review, we emphasize key contributions of the NHP model pre-clinical research for ZIKV, HCMV, HIV, IAV, *L. monocytogenes*, Ureaplasma species, and GBS. This work represents the foundation for development and testing of preventative and therapeutic strategies to inhibit infectious injury of human fetuses and neonates.

## Introduction

The teratogenic potential of pathogens was first realized in 1941, when Australian ophthalmologist Sir Norman McAlister Gregg reported the triad of congenital malformations (cataracts, heart disease, and hearing loss) of children born to mothers with a rubella virus infection in early pregnancy (Gregg, 1991). Vertical transmission from a maternal rubella virus infection is now known to also cause fetal glaucoma, microphthalmia, and developmental delay; the constellation of these symptoms is known as congenital rubella syndrome (Miller et al., 1982; Claus et al., 2020). By the late 20th century, additional pathogens like *Toxoplasma gondii*, human cytomegalovirus (HCMV), parvovirus B19, syphilis, herpes simplex virus (HSV), and varicella-zoster virus (VZV) were also identified as infectious teratogens transmittable to a fetus either *trans*-placentally or during delivery (Dudgeon, 1976). This list was recently expanded to include Zika virus (ZIKV), when an outbreak in northeastern Brazil in 2015–2016 was shown to be linked to a cluster of cases of neonatal microcephaly (Duffy et al., 2009; Cao-Lormeau et al., 2014; Cauchemez et al., 2016). As the ZIKV outbreak transformed into a global pandemic, the scientific literature linking maternal infection with fetal teratogenesis and stillbirth was strengthened by key studies in mice and non-human primates (NHP) (Adams Waldorf et al., 2016; Dudley et al., 2018; Waldorf et al., 2018).

Several animal models can be used to study infectious teratogenesis and fetal injury. When developing an animal model with an infectious disease, one must take into consideration microbial infection dynamics, species susceptibility, dose, route of inoculation, methods for assessing injury and translational relevance to humans. Animal models are necessary to perform critical studies of pathogenesis, clinical outcomes, and therapeutics, which cannot be performed in human pregnancies or infants. The NHP model is the closest animal model to human pregnancy and shares many similarities including placentation, pregnancy physiology, maternal-fetal interface, and timeline of fetal development (Furukawa et al., 2014; Grigsby, 2016; Stouffer and Woodruff, 2017). NHP models are also ideal for pre-clinical investigation of novel therapeutics and vaccines to prevent infectious disease. This review will focus on NHP models of teratogenesis, fetal and pediatric brain injury, preterm birth, stillbirth, and spontaneous abortion. We will also compare the advantages and disadvantages of NHP models to other animal models for the investigation of congenital and pediatric infectious injury. Although there are many infectious teratogens and pathogens with the potential to induce organ injury to fetuses and neonates, we will focus on several that have been studied in the NHP model including ZIKV, HCMV, human immunodeficiency virus 1 (HIV-1), influenza A virus (IAV), *Listeria monocytogenes*, and group B streptococcus (GBS). Finally, we feature novel advancements in testing vaccines and therapies in NHP models to prevent teratogenesis and injury, highlighting the translational potential to human pregnancy and neonatal care.

## Comparison of Animal Models of Teratogenesis

Animal models using pregnant mice, rats, guinea pigs, hamsters, and rabbits are often used to determine reproductive toxicity in pre-clinical testing and development of new drugs. Yet, these models differ significantly from women in both placentation and hormonal events surrounding parturition, limiting their translational impact – particularly for testing the efficacy of new therapeutics and vaccines (Table 1). While teratogenic phenotypes are observed in many animal models, species-specific differences in critical periods of fetal development also limit the range of investigable research questions. Nevertheless, animal models, especially NHP, provide a critical mechanism for exploring reproductive toxicity and teratogenesis (Table 2).

**TABLE 1:** Comparison of animal models for investigation of teratogenesis.

Characteristic	Human	Non-human Primate	Sheep	Guinea Pig	Rabbit	Rat	Mouse	Chick embryo	Zebrafish embryo	Xenopus embryo
Gestational length in days (mean ± SD)	280 ± 14	167 ± 7 (*M. mulatta*), 172 ± 7 (*M. nemestrina*)	144–151	60–70	31	21–23	19–21	N/A	N/A	N/A
Degree of development at birth	Precocial	Precocial	Precocial	Precocial	Altricial	Altricial	Altricial	N/A	N/A	N/A
Litter size or egg number (average)	1	1	1–3	2–4	5.5	8.2	10–12	1	Hundreds	Hundreds
Placenta (Furukawa et al., 2014)	Hemomono chorial, villous, discoid	Hemomo nochorial, villous, bidiscoid	Epithe liochorial cotyledonary	Hemomo nochorial, labyrinthine, discoid	Hemo dichorial, labyrinthine, bidiscoid	Hemo trichorial, labyrinthine, discoid	Hemo trichorial, labyrinthine, discoid	N/A	N/A	N/A
Uterus	Simplex	Bipartite	Bicornuate	Duplex uterus (two uterine horns, two cervices)	Duplex uterus (two uterine horns, two cervices)	Bicornuate	Duplex uterus (two uterine horns, two cervices)	N/A	N/A	N/A
Brain development (Clancy et al., 2007)	Extended brain development	Extended brain development		Brain growth spurt GD 45		Brain growth spurt PND 4–10		N/A	N/A	N/A
Teratogenesis Models	N/A	Human pathogens, biopharma ceuticals (e.g., monoclonal antibodies), subtle neurologic changes (Paule, 2005; Paule et al., 2011; Grant et al., 2019)	Useful for studying metabolic changes related to toxins	Useful for CNS toxicity evaluation because of extensive prenatal CNS development (Catlin et al., 1993)	Re commended non-rodent model for develop mental toxicity screening (Rovida and Hartung, 2009)	Re commended rodent model for develop mental toxicity screening (Rovida and Hartung, 2009)	Widely used in develop mental toxicology; particularly useful for gene × environment effects	Limb develop ment (Davey et al., 2018), mechanisms of toxicity	Rapid screen for chemicals and pharmace uticals, internal organs visible, transgenic models available	Rapid screen for chemicals and pharmac euticals [Frog embryo teratog enesis assay – Xenopus (FETAX)]
Advantages & Disadvantages	Directly translational, but limited to cross-sectional analysis	Directly translational, possible to do advance cognitive and behavioral testing, but expensive and limited in availability, high and variable pregnancy losses (Grossmann et al., 2020)	Chronically catheterized model is possible, need housing for large animals.	Similar placenta to humans. Because of extended gestation can evaluate late developmental effects by treating dam.	Low cost, small non-rodent	Low cost, must take litter effect into account (Jiménez and Zylka, 2021)	Low cost, small size, multiple inbred strains available, transgenic models available, must take litter effect into account (Jiménez and Zylka, 2021)	Low cost, physical mani pulations possible	Rapid 72–144 h test; 81% concor dance (Selderslaghs et al., 2012) with other species, not subject to animal use regulations	Rapid 96 h test; 75% concor dance (Fort and Paul, 2002) with other species, not subject to animal use regulations

**TABLE 2:** Comparison of similarities and differences with humans across common non-human primate models.

	Rhesus macaque (*M. mulatta*)	Pigtail macaque (*M. nemestrina*)	Cynomolgus macaque (*M. fascicularis*)	Common marmoset (*Callithrix jacchus*)	Olive baboon (*Papio anubis*)	Chimpanzee (*Pan troglodytes*)	African Green or Vervet monkey (*Chlorocebus aethiops*)
Reproductive cycle	28-day menstrual cycle	32-day menstrual cycle	28-day menstrual cycle	28-day estrous cycle	35-day menstrual cycle	35-day menstrual cycle	30-day menstrual cycle
Gestation length (days)	167	172	165	148	180	238	165
Seasonality	Breed in fall, give birth in spring/summer	No	No	No	No	No	No
Twinning	Uncommon	Uncommon	Uncommon	Common	Uncommon	Uncommon	Uncommon
Research advantages and disadvantages	Genomic data readily available (mgap.ohsu.edu/). Brain development well-documented (translatingtime.org/home)	Vaginal flora similar to humans (Patton et al., 1996). Vaginal epithelium less keratinized than *M. mulatta* (Hadzic et al., 2014). Year-round breeding.	Less expensive. Smaller, and can be housed in smaller cages. Commonly used in pharmaceutical research (Barrow, 2018).	Shorter gestation. Twinning common. Possible to make transgenics, and more practical because of shorter generation time.	Immunologically similar to human, expressing the four IgG subclasses seen in humans (Watts et al., 1999; Wolf et al., 2006). Large size of allows for use catheters, curettes, vaginal speculums, and dilators without modification (Nyachieo, Chai et al., 2007). Clear determination of reproductive cycle (Kling and Westfahl, 1978) phase due to large perineal sexual skin swelling (Bauer, 2015).	Genetically most related to humans, relevant for studying viral pathogenesis and vaccines. Endangered status, research highly restricted (Estes et al., 2018).	Well characterized maternal-fetal interface (Bondarenko et al., 2009; Dambaeva et al., 2009). Readily infected with SIV. Less expensive, easy to handle, accessible alternative to rhesus that is similar in behavior and physiology (Jasinska et al., 2013).

### Placentation, the Maternal-Fetal Interface

Placental permeability is determined by the structure and composition of the cell layers that separate maternal and fetal blood and varies among eutherian animals (Carter, 2020). The human placenta is hemochorial, with trophoblasts bathed directly in maternal blood; this thin maternal-fetal barrier makes oxygen and nutrient exchange highly efficient (Grigsby, 2016). Several animal species can model specific aspects of the human placenta. For example, the guinea pig has historically been an excellent animal model of human placental transfer and fetal growth restriction (Kelleher et al., 2011; Dyson et al., 2012; Morrison et al., 2018); the guinea pig hemomonochorial placenta deeply invades the decidua with proliferating trophoblast cells in a manner most similar to the human placenta (Kaufmann et al., 2003; Carter et al., 2006). Similarly, the sheep and its cotyledonary epitheliochorial placenta has a vascular structure similar to that of humans and can tolerate invasive procedures during pregnancy, making it a common model for studying fetal physiology and placenta vascular development (Grigsby, 2016). While these animals, and others, continue to make important contributions to an understanding of pregnancy and placentation, important differences in placental morphology (e.g., maternal-fetal interface, histological structure; Furukawa et al., 2014) have notable implications for studying teratogenesis.

Non-human primates have been used to model human implantation, placentation, parturition, and endometriosis (Grigsby, 2016). While placentation in NHP is characterized by generally superficial implantation and a less developed decidua lobe (Roberts et al., 2012), placental transfer in species like the rhesus macaque (*Macaca mulatta*) are analogous to the human placenta, making the pregnant NHP an ideal model for studying placenta permeability, pharmacodynamics, and toxicant transfer (Grigsby, 2016). Of note, complications in human pregnancy characterized by improper trophoblast invasion of the endometrium (e.g., preeclampsia, fetal growth restriction) currently lack a fully suited animal model (Carter and Pijnenborg, 2011; Carter, 2020).

### Timing of Fetal Development

Common laboratory animal models have contributed significantly to an understanding of fetal development. Researchers have used the chick embryo, frog, and zebrafish embryos and eggs to study the effect of teratogens and pathogens like ZIKV on the early precursors of the peripheral nervous system by loss of function analysis (Barriga et al., 2015; Narasimhan et al., 2020). Rabbit models have also been used to study the impact of factors like maternal dietary restriction and environmental pollutants on early embryonic and fetal-placental development (Fischer et al., 2012; Lopez-Tello et al., 2019; Carter, 2020). However, these animals and rodent models are altricial (Tran et al., 2000; Cronise et al., 2001), meaning that significant organ development occurs postnatally; this limits the translational ability of these models to study the temporal impact of infectious diseases on organogenesis and brain analogous to mid− to late human gestation. A precocial animal, born in a more advanced state of development (e.g., sheep, NHP; Grigsby, 2016; Carter, 2020), is better suited to evaluating the teratogenic potential of a pathogen on human fetal brain growth and differentiation, which largely occur in the third trimester of gestation (Dobbing and Sands, 1979). NHP models also offer the additional opportunity for researchers to assess deviations in neonatal behavior after birth (Nelson and Winslow, 2009; Bauman et al., 2014; Machado et al., 2015; Grant et al., 2019).

### Pathogen Sensitivity

Non-human primates most closely emulate the human immune system, an important consideration when analyzing the maternal-fetal-placental immune response to a pathogen (Safronetz et al., 2013). However, for many practical and scientific reasons, rodents and other animals are often used as the primary disease models (Table 1; Safronetz et al., 2013). For example, the pregnant mouse model is useful for investigating how the host immune system balances the need to maintain fetal tolerance with pathogen defense; the murine immune system is well characterized and research tools are commercially available (Lowe et al., 2018). Additionally, some animal models are natural hosts for the disease of interest, like adenoviruses for rodents and guinea pigs (Safronetz et al., 2013), and ZIKV for NHP (Narasimhan et al., 2020). However, many human pathogens need to be adapted to a specific animal model that may not adequately manifest human disease and pathology, like the mouse-adapted or guinea-pig-adapted Ebola virus (Safronetz et al., 2013). Similarly, *L. monocytogenes*, does not naturally infect the mouse gut and experimental modifications to change the method of inoculation or adapting humanized mice models sacrifice the integrity of placental infection (Lowe et al., 2018). Even among NHP, different species are not equally susceptible to all pathogens (Safronetz et al., 2013). The pigtail macaque (*Macaca nemestrina*) is known to be especially susceptible to multiple flaviviruses including dengue virus, Japanese encephalitis virus, chikungunya virus, hepatitis C, and other human pathogens (malaria, tuberculosis, chlamydia, Kaposi’s sarcoma) (Putaporntip et al., 2010; Bruce et al., 2013; Sourisseau et al., 2013; Nakgoi et al., 2014). Overall, the NHP represents an excellent model of human infectious disease.

Although NHP are often susceptible to human infectious diseases, the incidence of a teratogenic phenotype is often low in both humans and NHP models and may be highly dependent upon the gestational age at inoculation. For example, several species of NHP including African Green (or vervet) monkeys (*Chlorocebus aethiops*) (Sigurdardottir et al., 1963), patas monkeys (Erythrocebus patas) (Draper and Laurence, 1969), baboons (Horstmann, 1969), chimpanzees (Horstmann, 1969), and rhesus macaques (Parkman et al., 1965a) are susceptible to rubella virus, but rarely manifest clinical illness. In two studies challenging pregnant rhesus macaques with rubella virus in the first (Parkman et al., 1965b; Sever et al., 1966) and third trimesters (Parkman et al., 1965b), the classical findings of the congenital rubella syndrome were not observed; however, these studies were limited by small numbers [*N* = 4 (Sever et al., 1966), *N* = 6 (Parkman et al., 1965b)] and may not have captured infrequent events. In a third study that challenged rhesus macaques (*N* = 14) with rubella virus in the early first trimester, spontaneous abortion occurred in 9 of 14 (64%) pregnancies and congenital cataracts were observed in 2 of the 5 (40%) viable fetuses (Delahunt and Rieser, 1967). These studies demonstrate the challenge of studying teratogenesis in any animal model, which involves consideration of pathogen sensitivity, temporal susceptibility across gestation and the infrequent nature of some teratogenic phenotypes.

## NHP Models of Zikv Teratogenesis

### ZIKV-Associated Congenital Brain Injury in Humans and NHP Models

Zika virus is a mosquito-transmitted flavivirus recognized by the World Health Organization in 2016 as a “global public health emergency” after an outbreak in Brazil was associated with a surge in cases of congenital microcephaly and extensive brain injury (de Fatima Vasco Aragao et al., 2016; Gulland, 2016; Melo et al., 2016; Moura da Silva et al., 2016; Schuler-Faccini et al., 2016; Soares de Oliveira-Szejnfeld et al., 2016). Although a maternal ZIKV infection can result in a healthy neonate, the congenital Zika syndrome describes a set of classic central nervous system injuries including a massive reduction in the parenchymal volume of the brain, ventriculomegaly and abnormalities of cortical migration (Table 3; World Health Organization (WHO)., 2018a). Intracranial calcifications may occur at the gray-white matter junction, periventricular white matter, basal ganglia and/or thalami. ZIKV-induced cell death and dysregulated cell-cycle progression underlies abnormalities of brain gyral patterns that may meet diagnostic criteria for lissencephaly, polymicrogyria, or pachygyria. The cerebellum may also be abnormal, absent, or underdeveloped. In severe cases, skull collapse may also be present and is characterized by overlapping sutures with redundancy of skin folds. In addition to fetal brain injuries, ZIKV exposure *in utero* has been shown to lead to a variety of clinical outcomes including vision and hearing loss, contractures, brain lesions, and decelerated head growth months after birth (Franca et al., 2016; van der Linden et al., 2016; Aragao et al., 2017a,b; Honein et al., 2017; Petribu et al., 2017; Zin et al., 2017).

**TABLE 3:** Congenital anomalies potentially related to maternal ZIKV infection.

***Brain Abnormalities*** • Congenital microcephaly: HC < 3rd centile for gestational age and sex • Intracranial calcifications • Cerebral atrophy • Abnormal cortical formation (lissencephaly, polymicrogyria, pachygyria, schizencephaly, and gray matter heterotopia) • Corpus callosum abnormalities • Cerebellar abnormalities • Porencephaly • Hydranencephaly • Ventriculomegaly* or hydrocephaly* • Fetal brain disruption sequence (severe microcephaly, collapsed skull, overlapping sutures, scalp redundancy) • Other major brain abnormalities (thalamus, hypothalamus, pituitary, basal ganglia, or brainstem)
Neural Tube Defects and Other Early Brain Malformations** • Anencephaly or acrania • Encephalocele • Spina bifida without anencephaly • Holoprosencephaly or arhinencephaly
***Eye Abnormalities*** • Microphthalmia or anopthalmia • Coloboma • Congenital cataract • Intraocular calcifications • Chorioretinal anomalies (e.g., atrophy, scarring, macular pallor, retinal hemorrhage and gross pigmentary changes excluding retinopathy of prematurity) • Optic nerve atrophy, pallor and other optic nerve abnormalities
***Consequences of Central Nervous System Dysfunction*** • Congenital contractures (e.g., arthrogryposis, club foot, congenital hip dysplasia) with associated brain abnormalities • Congenital sensorineural hearing loss documented by postnatal testing

Table adapted from the US Centers for Disease Control standard case definition for birth defects potentially associated with ZIKV infection (World Health Organization (WHO)., 2018a). *Excludes isolated mild ventriculomegaly without other brain abnormalities or hydrocephalus due to hemorrhage. **Evidence for a link between ZIKV infections and neural tube defects is weaker than for other listed anomalies.

**FIGURE 1:**
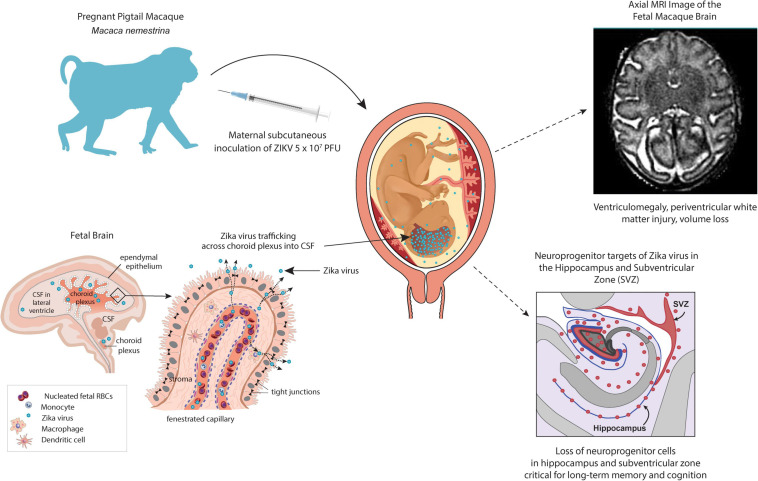
NHP pigtail macaque (*M. nemestrina)* model of the congenital Zika syndrome. A pregnant pigtail macaque is inoculated subcutaneously with 10^7^ plaque-forming units to mimic a mosquito bite leading to maternal viremia and detectable viral RNA in the fetus and fetal brain. A fetal magnetic resonance imaging scan demonstrates a periventricular T2-enhancing lesion **upper right**, which correlates with white matter injury. Neuroprogenitor cells (Tbr2+) in the hippocampus and subventricular zone (**lower right**, indicated by pink shading or pink dots) were significantly lower in ZIKV-exposed fetuses than in controls. In the **lower left panel**, an illustration of the choroid plexus demonstrates a hypothesized route for ZIKV entry into the fetal brain wherein the virus could directly enter the cerebrospinal fluid and subsequently, access all brain internal surfaces.

Non-human primates and murine models have been instrumental in providing the scientific evidence for maternal-fetal transmission, fetal injury and pathogenesis (Nguyen et al., 2017; Adams Waldorf et al., 2016, 2018a; Hirsch et al., 2018; Martinot et al., 2018; Mavigner et al., 2018b; Mohr et al., 2018). Many species of NHP have been shown to be susceptible to ZIKV and used as experimental models for ZIKV infection including the rhesus macaque (*Macaca mulatta*) (Abbink et al., 2016; Dudley et al., 2016; Aid et al., 2017; Coffey et al., 2017, 2018; Hirsch et al., 2017, 2018; Nguyen et al., 2017; Magnani et al., 2018; Martinot et al., 2018; Rayner et al., 2018), pigtail macaque (*Macaca nemestrina*) (Adams Waldorf et al., 2018a; O’Connor et al., 2018), cynomolgus macaque (*Macaca fascicularis*) (Koide et al., 2016; Schouest et al., 2020), olive baboon (*Papio anubis*) (Gurung et al., 2018; Gurung et al., 2020), chacma baboon (*Papio ursinus*) (Wastika et al., 2019), yellow baboon (*Papio cynocephalus*) (Buechler et al., 2017), and the malbrouck (*Chlorocebus cynosuros*) (Wastika et al., 2019), marmoset (*Callithrix jacchus*) (Chiu et al., 2017; Lum et al., 2018; Seferovic M. et al., 2018; Berry et al., 2019), black-tufted marmoset (*Callithrix penicillata*) (Terzian et al., 2018), tamarin (*Saguinus labiatus*) (Berry et al., 2019), squirrel monkey (*Saimiri* sp.) (Vanchiere et al., 2018), capuchin monkey (*Sapajus libidinosus*) (Favoretto et al., 2016; de Oliveira-Filho et al., 2018; Favoretto et al., 2019), and vervet or African Green monkey (*C. aethiops*) (Caldas-Garcia et al., 2020; Haddow et al., 2020). Animal models of adult and congenital ZIKV infection demonstrated broad viral tropism for cells and tissues throughout the body including the brain, spinal cord, placenta, testis/epididymis, ovary, uterus, multiple lymphoid tissues, joints, heart, and lungs (Adams Waldorf et al., 2016, 2018a; Chan et al., 2016; Cugola et al., 2016; Lazear et al., 2016; Ma et al., 2016; Miner et al., 2016; Mysorekar and Diamond, 2016; Sapparapu et al., 2016; Hirsch et al., 2017; Coffey et al., 2018; O’Connor et al., 2018). Studies in pregnant and neonatal NHP models revealed that ZIKV targets neuroprogenitor cells in the hippocampus (Adams Waldorf et al., 2018a; Mavigner et al., 2018b), a specialized brain region important for learning, memory, cognition and emotion/stress response (Spalding et al., 2013; Boldrini et al., 2018; Kempermann et al., 2018; Sorrells et al., 2018). A subcutaneous ZIKV inoculation in pregnant pigtail macaques led to a loss of fetal neuroprogenitor cells (neurogenic arrest) and disruption of neural circuitry in the dentate gyrus, a key hippocampal sub-region (Figure 1; Adams Waldorf et al., 2018a). In postnatal rhesus macaques, ZIKV infection led to hippocampal growth arrest and dysfunctional connectivity with other brain regions and abnormal socioemotional behavior in older animals (Mavigner et al., 2018b). Hippocampal injuries would be expected to correlate with early onset seizures/epilepsy in human infants, difficulties with memory development, and potentially late-onset depression and age-related cognitive decline in adulthood (Goncalves et al., 2016; Kempermann et al., 2018; Toda et al., 2018). Longitudinal studies are needed to understand how ZIKV-associated hippocampal injury in the fetus or young children might predispose to learning disorders, developmental delay, and/or neuropsychiatric disorders in childhood and adolescence (Adams Waldorf et al., 2018b). Indeed, NHPs can provide models for such longitudinal studies, as shown by a recent study describing auditory, ocular, brain structure, and neurobehavioral assessments of neonatal and infant rhesus monkeys delivered by cesarean section from dams receiving first trimester ZIKV inoculation (Koenig et al., 2020).

Impact of ZIKV on the CNS is not restricted to the brain. A high frequency of ocular malformations has been observed in infants with evidence of congenital ZIKV infection and microcephaly (Aleman et al., 2017). Ocular anomalies include macular pigment mottling, optic nerve hypoplasia, chorioretinal and iris coloboma, lens subluxation, retinal vascular abnormalities, cataracts, and maculopathy (Miranda et al., 2016; Ventura et al., 2016a, b,c,d; Agrawal et al., 2017; Honein et al., 2017; Yepez et al., 2017; Wong et al., 2019). Retinal defects include retinal thinning, discontinuity of the retinal pigment epithelium, and colobomatous-like excavation in the neurosensory retina, retinal pigment epithelium and choroid in multiple infants (Ventura et al., 2016d). Because of the pathway of retinal development from the neural tube, retinal lesions imply CNS damage even without brain abnormalities.

A pregnant rhesus macaque inoculated in the first trimester resulted in preterm premature rupture of membranes and fetal demise 49 days post infection (gestational day 95) (Mohr et al., 2018). Significant pathology at the maternal-fetal interface included acute chorioamnionitis and placental infarcts, and ZIKV RNA was disseminated throughout fetal and maternal immune system tissues. Fetal ocular pathology included a ventral choroidal coloboma, suspected anterior segment dysgenesis, and a dysplastic retina. Anterior segment dysgenesis refers to a spectrum of developmental anomalies resulting from abnormalities of neural crest migration and differentiation during fetal development (Gribnau and Geijsberts, 1985). An ocular coloboma is a congenital lesion associated with a failure in the closure of the embryonic (ocular) fissure causing defects of one or more ocular structures. In a previous study, optic nerve gliosis in two first trimester macaque infections, but did not identify other significant ocular pathology (Nguyen et al., 2017); thus the macaque model captures a wide disease spectrum of the impact of intrauterine infection on CNS development.

### Pathogenesis of ZIKV-Associated Fetal Brain Injury

The pathogenesis of congenital microcephaly associated with ZIKV infection during pregnancy is unknown, but clues from the histopathology in NHP models and human infants coupled with an understanding of fetal neuroanatomy and cortical migration suggests at least two routes to viral neuroinvasion (Figure 1). Once in the maternal bloodstream, ZIKV encounters the placenta with multiple permissible cell types that express putative viral receptors like Axl, Tyro3, and/or TIM1, which can facilitate viral entry, replication and eventual transmission to the fetus (El Costa et al., 2016; Jurado et al., 2016; Tabata et al., 2016; Pereira, 2018). In the fetal circulation, ZIKV would next encounter specialized barrier systems within the developing nervous system that regulate metabolite, gas, growth factor, and nutrient exchange (Spadoni et al., 2017). These barrier systems include the blood-cerebrospinal fluid (CSF) barrier within the choroid plexus, which regulates access to interior brain and spinal cord surfaces, as well as the blood-brain barrier (BBB) that regulates access to outer brain and spinal cord regions.

Evidence from NHP models indicates that ZIKV imparts fetal brain injury after crossing both the blood-CSF barrier and the BBB. The choroid plexus is a particularly important barrier and it is suggested that ZIKV passes through this network of cells to gain direct access to all internal brain and spinal cord surfaces (Figure 1, lower right panel) (Spadoni et al., 2017). The choroid plexus has been shown to be infected by chikungunya and dengue viruses (Lauer et al., 2018); ZIKV has now been shown to infect pericytes that line the blood vessels within the choroid plexus as well, suggesting this as one route of neuroinvasion (Kim et al., 2020). Periventricular injury patterns and ventriculomegaly in both humans and NHP models are consistent with the hypothesis that ZIKV can traffic across the choroid plexus to access the CSF lined by the ependyma (Adams Waldorf et al., 2016, 2018a; Melo et al., 2016; Chimelli et al., 2017; Sousa et al., 2017; Coffey et al., 2018; Martinot et al., 2018; Gurung et al., 2019; Nelson et al., 2020). Neuropathology demonstrated ependymal injury along the posterior lateral ventricles and periventricular white matter gliosis (Adams Waldorf et al., 2018a). Other ZIKV studies have demonstrated a similar neuropathology and pathology within the spinal cord (Coffey et al., 2018; Martinot et al., 2018; Gurung et al., 2019). ZIKV-associated ependymal injury could also impair cerebrospinal transport leading to ventriculomegaly (and rarely hydrocephalus), which has been observed in both NHP and human studies (Marques et al., 2019). ZIKV also likely traffics across the BBB to contribute to a complex array of human fetal brain injuries (Hazin et al., 2016; Chimelli et al., 2017), a hypothesis that is further supported by findings of hippocampal injury in the NHP model (Adams Waldorf et al., 2018a), which is a neurogenic niche not in direct contact with the CSF (Figure 1).

Zika virus exposed fetal NHP exhibited a striking loss of neurogenic output in the dentate gyrus subgranular zone (Adams Waldorf et al., 2018a). Immunohistochemistry revealed that while dentate gyrus NSCs (Sox2+ cells) were still present, the NSCs were highly disorganized due to ZIKV exposure and intermediate progenitors (Tbr2+/EOMES+ cells) were significantly reduced, indicating neurogenic arrest (Adams Waldorf et al., 2018a). Remaining newborn neurons (Doublecortin + cells) were dysmorphic and the entire granule cell layer was disrupted (Adams Waldorf et al., 2018a). Another report using a different ZIKV strain, and species (baboon) also found a reduction in fetal dentate gyrus Sox2 + NSCs (Gurung et al., 2019). Moreover, postnatal ZIKV infection in 5-week-old rhesus macaques also resulted in progressive hippocampal volume reduction and functional connectivity suggesting that infection in young children may produce hippocampal injury (Mavigner et al., 2018b). Longitudinal follow up at 12 months of age further revealed a reduced size of the amygdalae and underdeveloped dendritic branching of immature amygdala neurons following postnatal ZIKV infection, structural changes that coincided with amygdala functional connectivity differences, heightened emotional reactivity, and decreased prosocial behaviors (Raper et al., 2020). Overall, congenital ZIKV infection represents a complex series of events beginning with viral infection, subversion of host immunity and trafficking across multiple placental and fetal tissue barriers. Subversion of maternal-placental-fetal innate immunity is a fundamental event that facilitates ZIKV infection, viral propagation, and spread to the fetus (Nelson et al., 2020). ZIKV neutralizing antibodies can be detected in blood samples collected at birth in both NHP and human neonates, with higher levels of inflammatory cytokines found in the CSF and peripheral blood compared to the maternal counterpart (Adams Waldorf et al., 2016; Melo et al., 2016; Hirsch et al., 2018). The gestational timing of the maturation of innate immune antiviral pathways within fetal tissues and cell types is unknown and warrants focused research.

### ZIKV-Associated Spontaneous Abortion and Stillbirth

Several human and animal studies indicate that maternal ZIKV infections can cause spontaneous abortion and stillbirth (Miner et al., 2016; Schaub et al., 2016; van der Eijk et al., 2016; Coffey et al., 2018; Dudley et al., 2018; Hoen et al., 2018; Magnani et al., 2018; Mohr et al., 2018; Seferovic C.S. et al., 2018). Rates of ZIKV-associated pregnancy loss after 20 weeks have been reported to range between 1 and 2% (Brasil et al., 2016; Hoen et al., 2018; Walker et al., 2018), which represents a 10–20 fold greater rate than in healthy pregnant women (Wilcox et al., 1988). It is difficult to estimate the precise contribution of ZIKV infection to spontaneous abortion and stillbirth due to inconsistent reporting and data collection (Blencowe et al., 2016). In NHP infected with ZIKV in early gestation, miscarriage and stillbirth occurred at a 3–7 fold higher rate than in comparably housed healthy, ZIKV-unexposed pregnant macaques (Dudley et al., 2018). Although the mechanism for stillbirth is unknown, one NHP study indicates that placental injury and infarctions may compromise fetal oxygenation contributing to neural ischemia (Hirsch et al., 2018). A murine study indicated that type I interferon, a key antiviral defensive modulator, can alter placental development and trigger fetal death in the context of ZIKV infection (Yockey et al., 2018). The link between spontaneous abortion, stillbirth and infectious disease can be challenging in human studies due to the high baseline rate of spontaneous abortion, particularly with advanced maternal age. In the case of ZIKV, the link between experimental inoculation and spontaneous abortion and stillbirth was clearer than in human studies due to the comparatively low rate of spontaneous abortion in unexposed NHPs (Dudley et al., 2018).

### NHP Models of ZIKV: Conclusion and Therapeutics

At least 3,700 cases of congenital anomalies were reported in association with a maternal ZIKV infection, as of January 2018 (World Health Organization (WHO), 2018b). Molecular mechanisms of ZIKV and many other teratogenic pathogens induce congenital anomalies, fetal or neonatal injury remain poorly understood, but indicate a scientific need to better understand mechanisms of placental and fetal infection and development of fetal immunity. NHP models of ZIKV infection have played a critical role in developing and identifying numerous promising candidates for vaccines and therapeutics, which include various platforms like live-attenuated vaccines, inactivated virus vaccines, nucleic acid vaccines, and more (Abbink et al., 2016; Dowd et al., 2016; Osuna and Whitney, 2017; Pardi et al., 2017; Medina et al., 2018). In the wake of a dramatic reduction in ZIKV transmission after the 2016 epidemic, the challenge of enrolling enough volunteers to confirm the efficacy of vaccine candidates in phase III trials remains (Barrett, 2018; Poland et al., 2019; Castanha and Marques, 2020). With the potential for another ZIKV epidemic in the next couple decades (Castanha and Marques, 2020), it is imperative to continue development of ZIKV vaccines and therapeutics for pandemic preparedness.

## Cytomegalovirus Infection in Human and NHP Models

Human cytomegalovirus (HCMV) is a common β-herpesvirus that spreads through bodily fluids such as blood, saliva, and urine as well as through organ transplantation, breast milk and through vertical transmission to a fetus. Though often asymptomatic after the initial 2-to-3-week illness, CMV infections can persist as a latent or chronic infection late into adulthood. As the most common congenital infection globally (1 in ∼200 children), congenital HCMV (cCMV) infection is a frequent cause of infant neurological sequelae, including sensorineural hearing loss (SNHL) and cognitive or motor deficits (Yow and Demmler, 1992; Dollard et al., 2007; Kenneson and Cannon, 2007; Manicklal et al., 2013). Yet, despite the significant global impact of cCMV, there is not currently an effective vaccine to prevent HCMV acquisition or *in utero* infection. Several animal models have been applied to investigate CMV pathogenesis and transmission, including rodents [e.g., mice (Rawlinson et al., 1996), rats (Vink et al., 2000), and guinea pigs (McGregor et al., 2004)] and NHP [e.g., chimpanzees (Davison et al., 2003), rhesus macaques (Hansen et al., 2003), and cynomolgus macaques (Ambagala et al., 2011; Marsh et al., 2011)]. However, since CMV variants are highly species-specific, these models rely on CMV strains distinct from HCMV. Nevertheless, NHP CMV genomes most closely match that of HCMV.

### Functional and Genomic Similarity in Human and Rhesus Macaques

The similarity between HCMV and rhesus macaque cytomegalovirus (RhCMV) at the genome and amino acid sequence level makes rhesus macaques an ideal NHP model to investigate CMV infections. The size of the HCMV genome (229,354 bp) and RhCMV genome (strain 68-1; 221,459 bp) is comparable in length and the two genomes share 97% similarity at the nucleotide level. With regard to open reading frames (ORFs), both HCMV and RhCMV genomes are colinear and have approximately 250–260 potential ORFs. Eighty percent of the RhCMV ORFs are homologous to HCMV ORFs, and more than 90% of the RhCMV ORFs has an ortholog in HCMV genome at the protein family level (Hansen et al., 2003; Murphy et al., 2003; Rivailler et al., 2006; Oxford et al., 2008; Malouli et al., 2012; Stern-Ginossar et al., 2012).

### Congenital CMV Transmission in Human and Rhesus Macaque NHP Models

**FIGURE 2:**
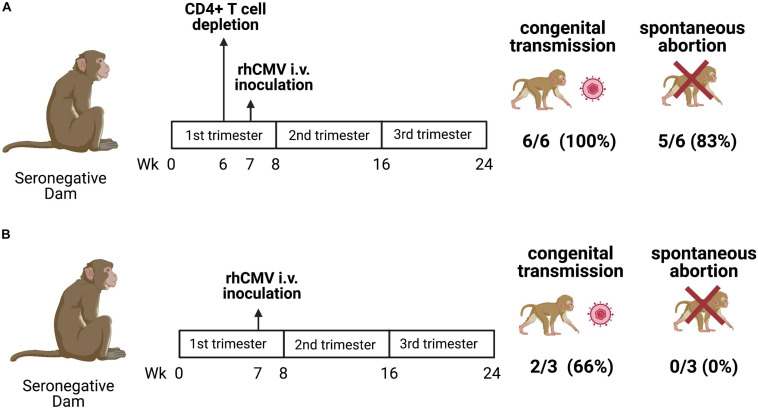
cCMV transmission in a CD4+ T cell-depleted and immunocompetent rhesus macaque (*M. mulatta*) NHP model. **(A)** Seronegative dams were CD4+ T cell-depleted at gestational week 7 and inoculated with RhCMV 1 week after CD4+ T cell depletion. All (6/6; 100%) dams transmitted RhCMV vertically and a spontaneous abortion occurred in 5 of 6 (83%) dams. **(B)** Immunocompetent seronegative dams were inoculated with RhCMV at week 7 of pregnancy. Vertical transmission occurred in two of three (66%) pregnancies with no spontaneous abortions (Bialas et al., 2015; Nelson et al., 2017).

Rhesus macaques are also an ideal model to study cCMV transmission, because the pathogenesis of CMV infection of human and rhesus macaques is remarkably similar (Bialas et al., 2015; Itell et al., 2017; Roark et al., 2020). Resembling the high seroprevalence of HCMV infection globally (83%) (Zuhair et al., 2019), the seroprevalence of RhCMV infection is approximately 95–100% among animals studied in primate research centers (Swack and Hsiung, 1982; Jones-Engel et al., 2006). Moreover, the fetal sequelae of HCMV and RhCMV infection are similar, sharing common manifestations such as hearing impairment, microcephaly, and fetal loss (London et al., 1986; Tarantal et al., 1998; Chang et al., 2002; Barry et al., 2006; Cheeran et al., 2009; Bialas et al., 2015; Nelson et al., 2017). Both antepartum, intrapartum and postpartum transmission (via breast) milk are commonly observed in HCMV infection (Diosi, 1997; Hamprecht et al., 2001; Fowler et al., 2003; Kenneson and Cannon, 2007; Hamprecht and Goelz, 2017). In the rhesus macaque model, placental transmission was possible in immunocompetent dams, though it occurred consistently and was more severe when maternal CD4+ T cell were depleted. As shown in Figure 2, the cCMV transmission rate of rhesus macaques with CD4+ T cell depletion is 6 of 6 (100%) with 80% fetal loss, while that of the immunocompetent animals is 2 of 3 (66%) with no fetal loss (Bialas et al., 2015). Notably, this high rate of transmission and fetal loss in CD4+ T cell-depleted dams was shown to be prevented by administration of passive RhCMV-specific IgG infusion prior to inoculation (Nelson et al., 2017). These findings suggest that rhesus macaques are an ideal NHP model to investigate cCMV transmission, establishing the importance of both maternal antibodies and CD4+ T cells in transmission risk.

### Implications of RhCMV NHP Models for Vaccine Development

The genomic similarities between HCMV and RhCMV as well as the multiple shared characteristics between human and rhesus macaque cCMV models make rhesus macaques an ideal preclinical NHP model for vaccine development. Two glycoprotein complexes, glycoprotein B (gB) and pentameric complex gH/gL/Ul128/UL130/UL131 (PC), on the HCMV virion surface have been proposed as vaccine targets due to their ability to elicit neutralizing activity (Anderholm et al., 2016; Gardner and Tortorella, 2016). There is a 60% shared amino acid identity between HCMV and RhCMV gB, and RhCMV gB shares a similar role and structure as HCMV gB (Kravitz et al., 1997). HCMV gB/MF59 adjuvanted vaccine provided partial efficacy in phase II clinical trials (Zhang and Pass, 2004; Bernstein et al., 2016), and several current HCMV vaccine platforms have added the PC (Gerna et al., 2017). RhCMV gB has been expressed in modified vaccinia Ankara virus (MVA) vectors, DNA expression plasmids, as well as a soluble protein subunits and result in elicitation of neutralizing antibodies (Abel et al., 2011; Valencia et al., 2019). Subunits of PC are also conserved between HCMV and RhCMV (Hansen et al., 2003; Rivailler et al., 2006; Wussow et al., 2013), and potent neutralizing antibodies can be induced in rhesus macaques with RhCMV gB and PC-expressing MVA vectors (Wussow et al., 2013). In conclusion, the rhesus macaque is an excellent preclinical model to examine CMV pathogenesis, immunity, and vaccine efficacy due to the genetic similarity of RhCMV genome and the comparable pathogenesis of RhCMV infection.

## NHP Models of Neonatal Central Nervous System Injury by HIV

Human Immunodeficiency Virus 1 (HIV-1) is a retrovirus that evolved from similar immunodeficiency viruses in NHP that spread to humans in the early-to-mid 20th century. Despite a prolific eruption of cases across the globe throughout the 1970s and 1980s, rates of new infections have steadily decreased since 1999, although the goal to end the HIV epidemic remains elusive. Nearly 38 million people were reported to be living with HIV in 2019 with a recorded 1.7 million new infections (UNAIDS, 2020). HIV is considered a chronic infection; often only minor symptoms are reported in the first few months or years of illness as the virus slowly replicates throughout the body’s lymph nodes and blood stream. Gradual degradation of the adaptive immune system through targeted destruction of CD4+ T lymphocytes allows progression to the late phase of HIV disease, Acquired Immunodeficiency Syndrome (AIDS) (Naif, 2013). Inadequate prenatal care or maternal treatment during pregnancy can result in vertical transmission of HIV-1 from mother to fetus. In addition, postnatal HIV-1 transmission through breastfeeding now accounts for over half of new pediatric infections. Children perinatally infected with HIV-1 can experience a range of neurologic complications, some of which persist even during antiretroviral therapy (ART) when HIV replication is suppressed in peripheral blood (Chahroudi Wagner and Persaud, 2018). These complications include cognitive impairment, delays in motor development, neuropsychiatric diseases and progressive encephalopathy (Donald et al., 2014; Phillips et al., 2016). The exact mechanisms underlying neurological disease are not elucidated, but may include continued viral replication in the central nervous system (CNS), neurotoxicity from antiretroviral drugs or viral proteins, chronic neuroinflammation, or neural injury due to secondary opportunistic infections such as *Toxoplasmosis gondii*, hCMV or the John Cunningham virus (Yilmaz Price et al., 2008; Decloedt et al., 2015; Dickens et al., 2017). It is critical to understand neuropathogenesis in the setting of perinatal transmission, wherein exposure to viruses during the *in utero* or postnatal stages of rapid brain development can lead to neurologic impairment in childhood, adolescence, and into adulthood (Knickmeyer et al., 2008; Hamprecht and Goelz, 2017; Mavigner et al., 2018b).

### SIV CNS Infection and Injury in NHPs

Macaques infected with the simian immunodeficiency virus (SIV) are widely used in HIV-1 research and provide insight into CNS infection and injury in the pediatric population (Evans and Silvestri, 2013; Carryl et al., 2015; Kumar et al., 2016; Obregon-Perko et al., 2020a). SIV (and the related simian-human immunodeficiency virus, SHIV) DNA can be found in the brain of infant macaques by 2 days post-challenge, where the virus can persist despite suppressive ART (Amedee et al., 1995; Hessell et al., 2016; Avalos et al., 2017; Mavigner et al., 2018a; Obregon-Perko et al., 2020b). Histopathological findings in SIV-infected infant macaques resemble those seen in HIV-1-infected children and include decreased brain growth, perivascular infiltrates of mononuclear cells, mineralization of vessels in the basal ganglia, and proliferation of glial cells (Lane et al., 1996; Westmoreland et al., 1999). A recent body of work in neonatal and infant macaques showed dramatic demyelination and reductions in neuronal populations of the hippocampus, injuries that could explain the neurocognitive and neuromotor decline sometimes seen in children living with HIV-1 (Facchini et al., 2002; Ruel et al., 2012; Curtis et al., 2014; Carryl et al., 2017). Reports of encephalitis are rare in pediatric HIV-1 and SIV infection relative to juveniles and adults. One macaque study attributed this to differences in BBB permeability and CCR5 expression in the brain (Kure et al., 1991; Delery et al., 2019). Only about 30% of SIV-infected macaques and HIV-infected patients present with neuropathological lesions, creating a challenge for robust studies of CNS injury (Williams et al., 2008). However, macaque models of consistent and accelerated progression to CNS disease have been developed using neurotropic viral strains, paving the way for studies of shorter duration with fewer animals (Zink et al., 1997; Kinman et al., 2004). In the setting of treated infection and undetectable plasma viral loads in infant macaques, levels of SIV RNA and DNA in the brain have been shown to be similar to those found in untreated infection with high plasma viral loads (Mavigner et al., 2018a). This finding may be at least partially explained by limited CNS penetration of the reverse transcriptase and integrase inhibitors used to suppress viremia in this study (Mavigner et al., 2018a).

### Insights From NHP and Human Clinical Studies for Treating Neurocognitive Decline in HIV-1 Infection

Various classes, and doses, of therapeutic agents have been proposed and explored for their potential to prevent neurocognitive decline in HIV-1 infection (Omeragic et al., 2020). One strategy is to impede viral infection in the brain by increasing ART drug concentrations to therapeutic levels in the CNS. In support of this approach, neurologic impairment has been negatively associated with ART regimens containing drugs with high CNS penetrance (Gill et al., 2016). Alternatively, nanoparticles may be employed to target the delivery of low CNS penetrating ART drugs across the BBB (Gong et al., 2020). Challenges with ART-based approaches include the risk for neurotoxicity with increased drug concentrations and the limited efficacy of ART in myeloid cells, thought to be the main cellular reservoir of HIV-1 in the brain (Robertson et al., 2012; Asahchop et al., 2017; Avalos et al., 2017). Encouragingly, in some children, a gradual recovery in HIV-related neurocognitive impairment has been described without modification to a suppressive ART regimen (Innes et al., 2020). Resolving inflammation in the brain also holds promise as an immunomodulatory approach for treating neurocognitive decline. HIV-1 patients treated with cenicriviroc, a dual chemokine receptor antagonist, showed decreased soluble activation markers, which was associated with improved cognitive performance (D’Antoni et al., 2018). Similarly, SIV-infected macaques administered natalizumab, an antibody against the α_4_-integrin, had lower soluble myeloid activation markers and decreased neuronal injury (Campbell et al., 2014). In an HIV-mouse model, a JAK 1/2 inhibitor, baricitinib, reduced neuronal cell activation, and reversed cognitive dysfunction (Gavegnano et al., 2019). Baricitinib-treated mice also had lower viral burden in the CNS, though infection was not completely eliminated. Total elimination of the CNS viral reservoir may ultimately require a “shock and kill” strategy, whereby pharmacological agents are used to reverse latency and increase viral gene expression in infected cells, making them vulnerable to immune clearance (Gama et al., 2017; Singh et al., 2021). Clinical management of neurological disease is not yet optimized and continues to be an active area of investigation. It is also important to note that pharmacotherapy studies with investigational agents, similar to those described here, are generally lacking in perinatal HIV-1 infection, but needed to ensure candidate treatments will be successful in this age group.

### NHP Models of HIV-1: Conclusion

There is much to be learned about the neuropathologic events that cause or precede clinical manifestations of HIV-1 infections in neonates, children and adults. NHP models capture many aspects of human neurodevelopment and pediatric infection that will be valuable in enhancing the understanding of HIV-1 CNS injury in children. A key question in contemporary HIV research is whether complete eradication of virus inhabiting the CNS will be required to achieve an HIV cure (Chahroudi Wagner and Persaud, 2018). While challenging to address in an experimental setting, NHP models may provide essential opportunities to study this relationship, helping guide clinical trials in children designed to control or eradicate HIV-1 infection from the brain and other sites of viral persistence in the body.

## Influenza A Virus NHP Model of Fetal Brain Injury

Influenza A Virus (IAV) is an orthomyxovirus characterized by rapidly acquired mutations in its surface-proteins, hemagglutinin and neuraminidase, that allow for evasion of adaptive human immune responses (Peteranderl, Herold and Schmoldt, 2016). This process of antigenic drift underlies the need for annual revision and alterations of influenza vaccines. Antigenic shift, in which new viral strains emerge through genomic reassortment, has contributed to global influenza pandemics - the most recent of which followed the emergence of the 2009 H1N1 viral strain. Sequelae of the initial respiratory illness of IAV has made it one of the leading causes of death and morbidity among vulnerable populations in the U.S. Despite pregnant women representing a particularly high-risk cohort, they remain a largely understudied population and the impact of IAV on fetal health and development has yet to be fully determined.

Vertical transmission of IAV is thought to be rare, but has not been studied comprehensively (Raj et al., 2014). However, IAV has been isolated from both placental tissue and amniotic fluid, having reached the affect cells following infection of the uterine decidua. It has been posited that IAV favors decidual tissue for viral replication before spreading to the fetal chorion and amnion (Raj et al., 2014). This process of viral replication may also have a direct cytopathic effect on chorionic cells, contributing to influenza-associated pregnancy loss. Some studies suggest that fetal death may also be secondary to the maternal inflammatory response following initial infection (Rasmussen et al., 2008). Human epidemiologic studies have raised concern that IAV might act to subtly injure the fetal central nervous system and predispose to the development of neuropsychiatric disorders like schizophrenia decades after birth (Hare et al., 1973; Parker and Neilson, 1976; Machon et al., 1983; Mednick et al., 1988; Barr et al., 1990; Brown et al., 2009; Al-Haddad et al., 2019). In rodents, prenatal exposure to influenza viral infection results in neural abnormalities in the offspring, with reduced cortical thickness and hippocampal volumes (Fatemi et al., 2002, 2008; Nawa and Takei, 2006). Rodents infected with IAV have a different disease course compared to humans, and IAV’s impact on fetal development varies significantly between rodent models and their human counterparts, limiting the impact of this translational research. NHP, however, share a hemochorial placenta with their human counterparts and give birth to only one infant at a time, ensuring advanced brain maturation that mirrors brain development in human children. However, only one NHP study of IAV has been performed to evaluate the impact of infection on fetal development. In an influenza model in pregnant rhesus macaque with the goal of studying the impact of infection on the developing fetal brain, pregnant dams were inoculated with A/Sydney/5/97 (H3N2) in the early third trimester. The offspring, despite no evidence of direct viral exposure, were found to have a significant reduction in bilateral cortical gray matter (cingulate and parietal lobes) at 1 year of age (Short et al., 2010). This translational research underscores an urgency to understand the impact of IAV on fetal development and NHP models will need to continue to be at the center of the assessment of this disease. Understanding the viral-host factors driving enhanced maternal influenza disease and/or fetal brain injury in a NHP model will enable investigating the role of vaccines and therapeutics in preventing maternal and fetal injury.

## *Listeria monocytogenes* NHP Model of Spontaneous Abortion and Stillbirth

*Listeria monocytogenes* (Lm) is a facultative intracellular gram-positive food-borne bacterium that is widespread in the environment. Although classically associated with unpasteurized dairy products, soft cheeses and preserved meats and fish, recently outbreaks have been associated with produce such as cantaloupes, lettuce, caramel apples, and others (Garner and Kathariou, 2016). Most individuals infected with Lm have an asymptomatic or mild illness, but listeriosis can cause sepsis and mortality in immunocompromised individuals, the elderly and young children (Hof, 2003). Pregnant women are at particular risk with listeriosis, not manifesting as maternal illness, but instead as stillbirth. Maternal listeriosis results in an increased risk for miscarriage and stillbirth, and even if pregnancy proceeds to delivery of a live infant, neonatal listeriosis causes meningitis, sepsis, and death (Wadhwa Desai and Smith, 2017). The antecedents and sequelae of listeriosis in pregnancy, particularly acute events, are difficult to study in the clinical setting, because infection may be asymptomatic in pregnancy. While rodent models have been valuable for understanding aspects of the biology of listeriosis in pregnancy, those models are complicated by a crucial amino acid difference in mice that results in failure of CDH1 (*E*-cadherin) to bind internalin A on the bacterial surface – an interaction critical to the ability of Lm to invade host cells (Bonazzi et al., 2009).

**FIGURE 3:**
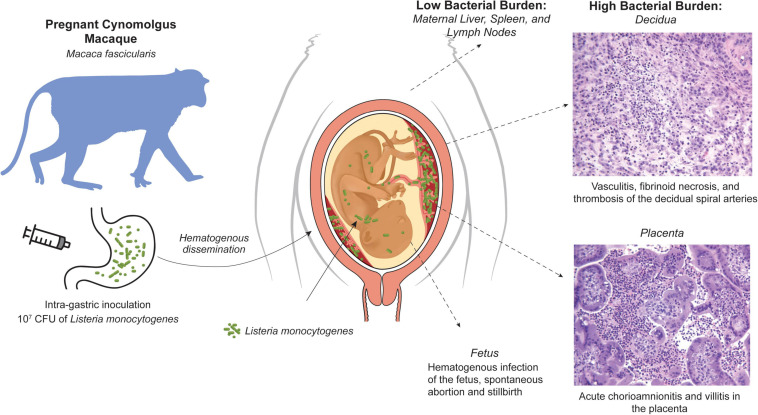
NHP cynomolgus macaque (*M. fascicularis*) model of listeriosis in pregnancy. Intragastric inoculation of *L. monocytogenes* in the first trimester results in bacteremia that is associated with a modest bacterial burden in classic Listeria target organs (liver, spleen, lymph nodes), but profound infection and histopathology at the maternal-fetal interface, fetal infection, and fetal demise.

The NHP model is an important adjunct with significant histological and functional relevance of the maternal-fetal interface, in particular the immune environment (Golos et al., 2010). Notably, cynomolgus macaques (*Macaca fascicularis*) have a well-characterized maternal-fetal interface making it a valuable Lm model for assessment of fetal impact (Bondarenko et al., 2009; Dambaeva et al., 2009). The risk of first trimester listeriosis is particularly difficult to address in human clinical cases, and macaques have been used in a series of studies of the impact of Lm inoculation on pregnancy (Figure 3; Smith et al., 2003; Smith et al., 2008; Wolfe et al., 2017; Wolfe et al., 2019). Intragastric inoculation in later gestation has previously been shown to result in an elevated risk for pregnancy loss or stillbirth, however there was no assessment of the bacterial burden or histopathology of the maternal-fetal interface or the fetus itself. Inoculation during the fifth and sixth week of gestation resulted in a very high incidence (∼80%) of fetal demise, as early as 7 days after maternal infection. Nearly all dams had bacteremia and fecal shedding that preceded fetal demise (Wolfe et al., 2017). The macaque model allowed surgical collection of both fetal and maternal tissues upon recognition of fetal demise. Examination of the bacterial burden in maternal tissues revealed, as expected, modest colonization of the usual target tissues in the dam liver, spleen and lymph nodes; remarkably, the bacterial burden in the decidua and the placental bed was 3–4 orders of magnitude higher. At the maternal-fetal interface, suppurative inflammation and necrosis in the decidua and vasculitis of the maternal spiral arteries was consistently noted with microabscesses in the extravillous cytotrophoblastic shell of the placenta, placental villous necrosis and suppurative intervillositis. Fetal membranes also displayed chorioamnionitis and there were gram-positive rods within the umbilical cord.

These studies demonstrated that in early pregnancy, there was significant risk to the fetus of rapid miscarriage. Fetal bacteria was widely disseminated indicating hematogenous spread, as would be inferred by the presence of inflammatory cells in the villous mesenchyme of the placenta (Wolfe et al., 2017); gram-positive tissues included the lung, liver, periosteum, perichondrium, and fetal cranium, as well as ocular muscles, neural tissue, and the developing skull. This profile of bacterial burden suggested that rapidly growing highly vascularized structures had greater risk for colonization (Wolfe et al., 2019). Interestingly, the highly efficient rate of vertical transmission and adverse pregnancy outcomes was not seen when maternal inoculation was done in the third trimester (Smith et al., 2008; Wolfe et al., 2019); although if an increased inoculum was used, 3 of 4 subjects inoculated early in the third trimester had fetal demise (Wolfe et al., 2019). The first trimester may thus be more sensitive to maternal listeriosis. In the early pregnancy decidua, there is active neovascularization and macrophage infiltration as decidual spiral arteries are remodeled by invading endovascular trophoblasts (Bondarenko et al., 2012). In this situation, a more robust immune response may be mounted in the decidua, whereby acute inflammation creates greater damage to the maternal fetal interface, allowing bacteria to cross the placental trophoblast barrier (Bondarenko et al., 2007; Bondarenko et al., 2012; Carter et al., 2015).

Histopathology of maternal-fetal interface tissues in third trimester pregnancy was scored as a sum of abnormal histological findings (Wolfe et al., 2019). Of significant interest was the presence of an elevated histopathological placental score, even in pregnancies that progressed to term without fetal loss or evidence of infection. Likewise, PCR analysis of inflammation markers assessed by Luminex assay demonstrated that Lm exposure, regardless of pregnancy loss or survival, clustered apart from control pregnancy. While this was a terminal study for the fetus, the developmental sequelae of infection impact on the placenta and decidua in the offspring is not known from these studies. The macaque model can be used for further studies to address the potential long-term impact on the infant, as is being done with macaque models of maternal Zika virus infection (Koenig et al., 2020).

With the expanding occurrence of listeriosis outbreaks due to contamination of the food supply, relevant animal models will be important to develop potential mitigation. While antibiotic therapy is available for pregnant women, the mother is often asymptomatic and thus disease progresses to threaten adverse pregnancy outcomes without the opportunity to intervene. Further research with NHPs may ultimately provide insight into the mechanisms that determine why adverse outcomes are more likely in the first trimester, or which specific factors allow early identification of potentially devastating early pregnancy infection and thus quicker antibiotic intervention. Other important inquiries might be addressed through NHP models as well; it is unclear whether vaccines based on an Lm vector backbone (Morrow et al., 2019) would provide additional protection during pregnancy or if such a vaccine would trigger sensitization and lead to a more profound impact on immune and inflammatory outcomes at the decidual interface. Other approaches, such as developing probiotic treatment (Corr et al., 2007; Ryan et al., 2021) may prove productive and should be evaluated in NHP models. It is also unknown whether pre-pregnancy exposure to Lm provides protection from adverse outcomes in pregnancy, or what genetic determinants of pathogenesis might target the maternal-fetal interface; these questions can be answered using the NHP model.

## NHP Model of *Ureaplasma parvum* Preterm Birth and Fetal Injury

Preterm labor and preterm birth is strongly associated with intrauterine infection during early gestation (Kelleher et al., 2020). Invasion of the amniotic cavity by *Ureaplasma parvum (U. parvum)* in particular triggers a strong inflammatory response, and has been implicated in neonatal sepsis, neurodevelopmental abnormalities, and bronchopulmonary dysplasia (Yoon et al., 1998; Novy et al., 2009; Waites et al., 2009). However, due to the polymicrobial nature of such intrauterine infections, the exact mechanism by which *U. parvum* induces inflammation has been difficult to elucidate in human clinical studies. The pregnant NHP model thus has been instrumental in elucidating the causal relationship between *U. parvum* infection, preterm labor, and the associated sequelae. In a chronically catheterized rhesus macaque, intraamniotic inoculation with clinical isolates of *U. parvum* serovar 1 resulted in upregulation of pro-inflammatory cytokines, prostaglandins and leukocytes in the amniotic fluid, chorioamnionitis, fetal pneumonia and increased uterine activity (Novy et al., 2009). Collectively, these results implicated *U. parvum* as an important pathogen to fetal lung injury and preterm labor (Novy et al., 2009). Studies of *U. parvum* infection in pregnant baboon models demonstrated similar findings (Walsh et al., 1993; Yoder et al., 2003). *U. parvum* inoculated into the amniotic fluid was also associated with decreased neonatal brain growth and white matter maturation in a rhesus macaque model (Kelleher et al., 2017).

Whether antibiotics to treat a *U. parvum* infection *in utero* might prevent preterm birth and fetal injury was unclear from clinical studies (Grigsby et al., 2012). The pregnant NHP model provides the valuable opportunity for researchers to study antenatal antibiotic therapy under controlled experimental conditions. Indeed, maternal administration of azithromycin has been demonstrated to inhibit preterm uterine contractions, minimize fetal lung injury, and improve fetal hemodynamics due to *a. parvum* intraamniotic infection in the chronically catheterized rhesus macaque (Grigsby et al., 2012; Kelleher et al., 2020). Moving forward, NHP studies will further inform current understanding of the pathobiology of *U. parvum* and other bactrerial intra-amniotic infections.

## Group B Streptococcus NHP Model of Preterm Birth and Fetal Injury

**FIGURE 4:**
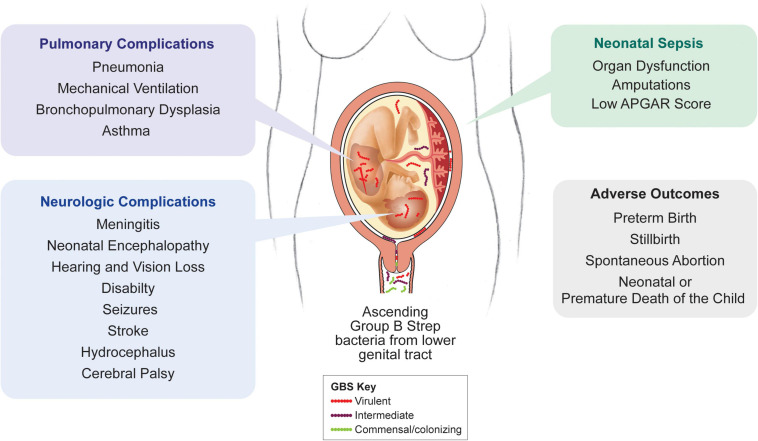
Clinical outcomes of invasive GBS infection. In humans, invasive GBS infections can result in a spectrum of obstetrical and neonatal systemic, pulmonary, and neurological complications. The majority of these complications are also observed in the NHP model.

Group B streptococcus is a gram-positive, chain-forming bacterium that colonizes the rectovaginal tract of about 20% of women worldwide (Russell et al., 2017). Vaginal colonization is generally asymptomatic, but GBS invasion into the placenta, amniotic cavity and fetus can lead to severe complications during pregnancy. Annually, GBS is responsible for at least 409,000 cases of invasive disease in infants, 147,000 cases of stillbirth or infant deaths, and up to 3.5 million cases of preterm labor (Figure 4; Seale et al., 2017). Furthermore, invasive GBS infection can cause fetal injury associated with a number of chronic sequelae (Seale et al., 2017). Studying the underlying mechanisms through which GBS exerts these outcomes is challenging. GBS serotype prevalence varies greatly by region, making it difficult to compare cases and outcomes across human cohorts (Madrid et al., 2017; Russell et al., 2017). Further, bacterial invasion of the amniotic cavity and fetal compartments occurs silently; investigating the pathogenesis is not possible until there is a fulminant infection of the placenta or amniotic cavity. Studies in pregnant women have thus underscored the inflammatory cascade associated with GBS and other bacterial infections but fell short of elucidating the early events of pathogenesis. And as previously discussed, other mammalian models do not adequately recapitulate important characteristics of human pregnancy, further complicating this effort (Adams Waldorf et al., 2011b).

**FIGURE 5:**
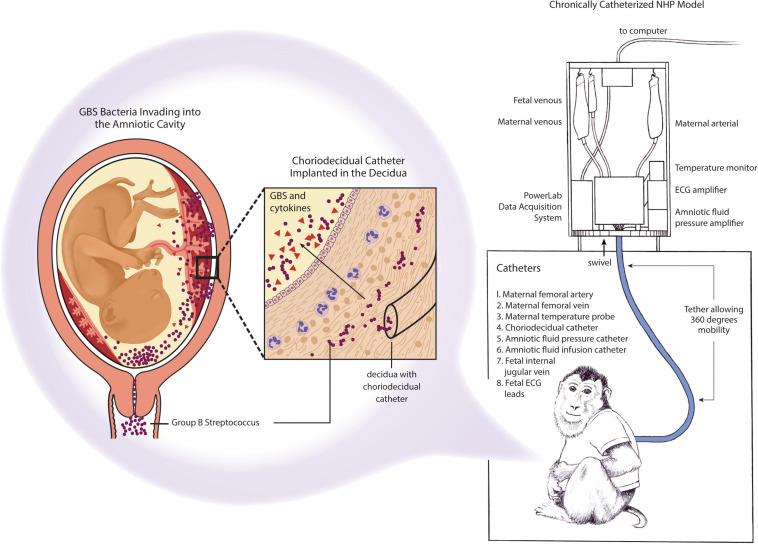
Chronically catheterized pregnant NHP model of GBS infection and preterm labor. Catheters are also inserted into the dam (maternal vein), amniotic fluid to monitor pressure and enable inoculations and into the fetus (fetal vein). ECG leads may also be placed in the fetal chest wall and arm to monitor the fetal heart rate. The choriodecidual catheter is inserted in between the uterus and the chorioamniotic membranes of the lower uterine segment (within the decidua) of the pigtail macaque (*M. nemestrina*). Inoculation of GBS through the choriodecidual catheter in the lower uterine segment simulates an ascending infection of bacteria from the vagina, which is hypothesized to occur in human cases of preterm labor induced by GBS or other bacteria.

To overcome these challenges, a chronically catheterized pregnant NHP model was developed to monitor GBS-induced disease progression, immune response, and pregnancy outcomes following an experimental GBS inoculation (Gravett et al., 1994; Adams Waldorf et al., 2011a; Boldenow et al., 2016; Coleman et al., 2020, 2021). This model is highly translational based on several characteristics: (1) inoculation of GBS in the choriodecidual space where GBS is hypothesized to first invade the placenta, (2) serial sampling of maternal and fetal blood and amniotic fluid, (3) monitoring of uterine contractions and fetal heart rate over time, and (4) administration of antibiotics or immunotherapeutics to prevent preterm birth or fetal injury (Figure 5). This data can be used to delineate the timing of microbial invasion of the amniotic cavity and subsequent fetal infection relative to the progression of adverse outcomes. Host-pathogen responses, innate immunity and –omics approaches to systems biology can also be applied to the study of preterm birth and fetal injury.

### Designing a Model of GBS-Induced Preterm Labor Model

Early studies of infection-associated preterm birth were performed in rhesus macaques. The first study in the NHP inoculated GBS directly into the amniotic fluid and observed elevated interleukin-1 beta (IL-1β), tumor necrosis factor-alpha (TNF-α), and interleukin-8 (IL-8), followed by preterm labor (Gravett et al., 1994). These experiments confirmed that a bacterial infection could cause preterm birth and underscored the importance of cytokines in preterm labor. To determine if cytokines could induce preterm labor, experimental inoculations of IL-1β, TNF-α, IL-6, and IL-8 were performed in the chronically catheterized NHP model. Inoculation of IL-1β or TNF-α induced preterm labor, while IL-6 or IL-8 did not (Sadowsky et al., 2006). The model was later modified to inoculate GBS into the choriodecidual space of the lower uterine segment, a site that was hypothesized to represent the first site that GBS might infect the placenta. Using this new route of inoculation, low-dose GBS (10^2^–10^4^ cfu/mL) caused modest TNF-α production with moderate amniotic fluid prostaglandins and mild uterine activity and did not invade the amniotic cavity. In contrast, GBS administered at a higher dose (10^3^–10^6^ cfu/mL) induced dramatic elevations in pro-inflammatory cytokines and invaded the amniotic cavity and fetus (Grigsby et al., 2010). Collectively, these studies culminated in the development of an NHP model whereby choriodecidual inoculation of pregnant animals with GBS leads to preterm labor. The next step was to interrogate how choriodecidual and intraamniotic inflammation, as well as GBS bacterial burden, may influence other adverse outcomes, such as placental and fetal injury.

### Interrogating GBS-Associated Fetal Lung and Placental Injury

During pregnancy, invasive GBS infection can cause significant fetal lung, cardiac, and brain injury, which are each associated with lifelong sequelae (Seale et al., 2017). In addition to defining GBS-induced preterm labor mechanisms, the GBS NHP model is poised to interrogate GBS-induced fetal injury due to the extensive array of fetal samples collected at necropsy, including the lung, brain, and heart. In an initial study of GBS choriodecidual infection in pigtail macaques, preterm labor occurred in 2 of 5 (40%) animals, but histologic evidence of fetal lung injury occurred in 4 of 5 (80%) (Adams Waldorf et al., 2011a). Specifically, these fetal lungs demonstrated neutrophil and macrophage infiltrates and thickened alveolar septa by histology. The most severe case of fetal lung injury also had high IL-6 in fetal plasma, which meets the human clinical criteria for fetal systemic inflammatory response syndrome. Surprisingly, GBS was only recovered at the membrane inoculation site of two monkeys (one of which experienced preterm labor) and all animals were culture negative for GBS in the amniotic fluid (Adams Waldorf et al., 2011a). In addition, fetal lung injury was observed in the absence of preterm labor or chorioamnionitis. Thus, fetal lung injury may occur even in the absence of GBS invasion into the amniotic fluid suggesting that inflammation is sufficient to induce this outcome. In a microarray study of these fetal lung tissues, key developmental gene sets in the fetal lung were downregulated (angiogenesis, morphogenesis, and cellular differentiation) in tandem with the innate immune response (McAdams et al., 2012). This data was the first to suggest a transcriptional signature of the fetal origins of bronchopulmonary dysplasia, a neonatal condition of prematurity characterized by simplified airways that failed to complete development.

A fulminant GBS infection of the chorioamniotic membranes was known to cause chorioamnionitis, an inflammation of the placental membranes associated with preterm labor. However, the effect of an early, cleared GBS infection on placenta integrity and the placental innate immune response was unknown. Microarray analysis of the chorioamniotic membranes obtained from NHPs following GBS clearance revealed that exposure to GBS resulted in downregulated production of multiple cytokeratin and collagen genes, components of amniotic epithelial tensile strength. Transmission electron microscopy demonstrated cytokeratin retraction and loss of intermediate filaments (Vanderhoeven et al., 2014).

### GBS Virulence Factors and Their Roles in Preterm Labor and Fetal Lung Injury

These early GBS studies in the NHP identified temporal relationships between a transient choriodecidual infection, inflammation in the amniotic fluid, microbial invasion of the amniotic cavity, preterm labor and fetal lung injury. However, these studies utilized the wild-type GBS serotype III ST-17 strain known as COH1; this strain is clinically relevant, but does not display high expression of certain well-described GBS virulence factors such as the hemolytic pigment and the secreted hyaluronidase enzyme (HylB), both of which are linked to GBS-associated preterm labor in mouse models (Whidbey et al., 2013; Vornhagen et al., 2016). Therefore, to better appreciate GBS mechanisms driven by these factors and their role in preterm labor, additional GBS strains were used that either overexpressed the hemolytic pigment (Whidbey et al., 2013) or HylB in the NHP model (Gendrin et al., 2018).

Group B streptococcus strains that overexpress GBS hemolytic pigment have a mutation in *covR*, a two-component system response regulator, and have been isolated from women in preterm labor (Whidbey et al., 2013). In a preterm NHP model, choriodecidual inoculation of hyperpigmented GBS COH1Δ*covR* resulted in more efficient penetration of the placental chorioamniotic membranes and subsequent infection of the fetus. Microbial invasion of the amniotic cavity was associated with chorioamnionitis. However, despite the significant influx of neutrophils to the site of infection, hyperpigmented GBS subverted primary host defense mechanisms by inducing pigment-mediated neutrophil cell death and resisting neutrophil extracellular traps, which ultimately promoted fetal sepsis and preterm labor (Boldenow et al., 2016). Preterm labor was associated with the fetal inflammatory response syndrome, marked by elevated fetal IL-6 in the plasma. Interestingly, GBS-associated fetal inflammatory response syndrome was also associated with elevated IL-6 and IL-8 levels and a greater bacterial burden but minimal neutrophil infiltration in fetal cardiac tissue. In these animals, gene sets involved in fetal heart development, such as cardiac morphogenesis and vasculogenesis gene networks, were downregulated; premature arrest of the fetal cardiac development program may contribute to cardiac dysfunction later in life (Mitchell et al., 2018). Collectively, these findings suggest that inflammation at the maternal-fetal interface is ineffective in curtailing fetal bacteremia, fetal injury and preterm labor in the context of infection with hyperpigmented, hypervirulent GBS.

In contrast to hyperpigmented GBS strains, clinical isolates of non-pigmented GBS with increased hyaluronidase activity have also been associated with adverse pregnancy outcomes and neonatal infection (Vornhagen et al., 2016). GBS HylB breaks down hyaluronan (HA) to into HA disaccharides that block recognition and subsequent signaling by toll-like receptor (TLR) 2 and TLR4 (Kolar et al., 2015). NHP infected with GBS that highly express HylB (strain GB37; Gendrin et al., 2018) consistently exhibited microbial invasion of the amniotic cavity, fetal sepsis and preterm labor (Kolar et al., 2015; Coleman et al., 2021). Notably, infection with GB37 yielded delayed cytokine responses at the choriodecidual membranes, despite rapid invasion of the amniotic cavity and neutrophil and CD8+ T recruitment; this is starkly in contrast to the rapid proinflammatory responses associated with infection by hyperpigmented GBS. Despite significant immune cell infiltrate to the maternal-fetal interface, digital spatial profiling of the uterine mucosa in GB37-infected NHP revealed that even in tissues where GBS had been detected, inflammation-associated markers were dampened relative to baseline. GB37 also blunted ROS production by neutrophils in a TLR2/4 dependent manner, suggesting that GBS uses HylB to evade host immune recognition by blocking TLR2/4 signaling and downstream ROS production in neutrophils (Coleman et al., 2021). In combination with the prior work on hyperpigmented GBS strains (Boldenow et al., 2016), this study highlights that a chronically catheterized NHP model can demonstrate the diverse roles of various GBS virulence factors in disease pathogenesis that would otherwise be difficult to discern in other animal models.

### Testing Preterm Labor Therapeutics in the GBS NHP Model

As IL-1β and TNF-α are strong inducers of preterm labor (Sadowsky et al., 2006), inhibition of chemokine and cytokine responses is an attractive therapeutic approach for the prevention of GBS-induced preterm labor. In pregnant NHP, pre-treatment with a broad-spectrum chemokine inhibitor dampens pro-inflammatory cytokine levels, such as IL-8 in the maternal plasma and amniotic fluid, and IL-6, IL-1β, and IL-7 in fetal tissues. Although prophylactic administration of a broad spectrum chemokine inhibitor decreased the frequency of GBS-induced uterine contractility and preterm labor, it failed to protect the fetus from adverse sequelae such as microbial invasion of the amniotic cavity and fetal bacteremia. Suppression of pro-inflammatory cytokines led to uncontrolled bacterial replication and dissemination, causing increased neutrophil and macrophage infiltration to chorioamniotic membranes and fetal lungs (Coleman et al., 2020). These findings suggest that a better strategy would likely be to utilize immunomodulators in combination with antibiotics to allow control of both GBS replication and the pro-inflammatory immune response.

In the NHP model, the combination of antibiotics and immunomodulators was tested in the context of an amniotic fluid inoculation of GBS. In these experiments, uterine activity was closely monitored after GBS inoculation. Once uterine activity doubled over a 2-h period, a combination of ampicillin with and without dexamethasone and indomethacin was administered. Although ampicillin treatment extended gestation by a few days, ampicillin plus immunomodulators prolonged gestation by a full week in 4 of 5 (80%) monkeys. Both treatments were successful in eradicating, or drastically reducing recovery of, GBS from AF and fetal blood, meninges, and lung. Immunomodulators with ampicillin therapy was also associated with a reduction in IL-1β in the amniotic fluid and fewer neutrophils in the chorioamniotic membranes (Gravett et al., 2007). These studies highlight the translational strength of this model.

## Conclusion

We remain unprepared to protect pregnancies from many teratogenic viral threats, as well as from other emerging pathogens with teratogenic potential. The expanding landscape of emerging pathogens with an unknown potential to induce teratogenesis or fetal injury necessitates a dedicated scientific effort to understand the mechanisms of disease pathogenesis and host response in pregnant and neonatal animal models. Many animal models have provided a critical foundation on which to assess disease pathogenesis, host pathogen response, and teratogenic fetal impact. However, the limited translational capacity of most mammalian models to human pregnancy has brought the NHP model into particular focus. This review demonstrates the critical utility of the NHP model in the study of a select few teratogenic pathogens: ZIKV, HCMV, HIV-1, IAV, Lm, and GBS. The notable similarities between humans and NHPs including the maternal-fetal interface, fetal development and pathogen sensitivity make the NHP ideal for the study of infectious disease and therapeutics in pregnancy. NHP models are essential to determining the potential for fetal injury and teratogenesis due to new pathogens and the efficacy of therapeutics to prevent fetal damage.

## Author Contributions

All authors listed have made a substantial, direct and intellectual contribution to the work, and approved it for publication.

## Conflict of Interest

SP is a consultant for Merck, Moderna, Pfizer, and Dynavax vaccines, and collaborates with Merck and Moderna on sponsored research programs around CMV vaccines. The remaining authors declare that the research was conducted in the absence of any commercial or financial relationships that could be construed as a potential conflict of interest.
